# Intelligent Recognition of Muffled Blasting Sounds and Lithology Prediction in Coal Mines Based on RDGNet

**DOI:** 10.3390/s25247601

**Published:** 2025-12-15

**Authors:** Gengxin Li, Hua Ding, Kai Wang, Xiaoqiang Zhang, Jiacheng Sun

**Affiliations:** School of Mining Engineering, Taiyuan University of Technology, Taiyuan 030024, China; 2023521220@link.tyut.edu.cn (G.L.);

**Keywords:** muffled blasting sounds, acoustic emission, lithology prediction, multimodal deep learning, RDGNet, intelligent monitoring, roadway surrounding rock failure

## Abstract

In the Yangquan coal mining region, China, muffled blasting sounds commonly occur in mine surrounding rocks resulting from instantaneous energy release following the elastic deformation of overlying brittle rock layers; they are related to fracture development. Although these events rarely cause immediate hazards, their acoustic signatures contain critical information about cumulative rock damage. Currently, conventional monitoring of muffled blasting sounds and surrounding rock stability relies on microseismic systems and on-site sampling techniques. However, these methods exhibit low identification efficiency for muffled blasting events, poor real-time performance, and strong subjectivity arising from manual signal interpretation and empirical threshold setting. This article proposes retentive depthwise gated network (RDGNet). By combining retentive network sequence modeling, depthwise separable convolution, and a gated fusion mechanism, RDGNet enables multimodal feature extraction and the fusion of acoustic emission sequences and audio Mel spectrograms, supporting real-time muffled blasting sound recognition and lithology classification. Results confirm model robustness under noisy and multisource mixed-signal conditions (overall accuracy: 92.12%, area under the curve: 0.985, and Macro F1: 0.931). This work provides an efficient approach for intelligent monitoring of coal mine rock stability and can be extended to safety assessments in underground engineering, advancing the mining industry toward preventive management.

## 1. Introduction

As the mining depth has increased, the stability of rocks surrounding deep roadways has emerged as a critical issue. In the Yangquan mining district of Shanxi Province, China, mining-induced low-frequency muffled blasting sounds are frequently emitted from within the coal-rock mass and are widely regarded as manifestations of stress concentration and microfracture propagation [1,2]. Detailed field monitoring at Yangsheng Coal Mine revealed that this deep site exhibits high in situ stress. Hence, these muffled blasting phenomena result from the abrupt release of accumulated elastic strain energy during the deformation and failure of the surrounding rock. This sudden energy release represents a common physical mechanism driving a continuum of mining-induced dynamic disturbances, from the muffled blasting sounds documented in the present study to severe rockbursts widely reported in deep coal mines worldwide [3,4,5]. Given that rockbursts, roof falls, and related dynamic disasters—many of which are preceded by similar acoustic precursors—remain a leading cause of fatalities and production losses in deep mining, the effective monitoring of such low-frequency acoustic signals is critical for early warning and safe production.

Surrounding rock stability is primarily analyzed based on manual inspections and using on-site sampling techniques, such as borehole coring and visual fracture assessment. However, these approaches suffer from low detection efficiency, poor timeliness, and high subjectivity. Under complex geological conditions, such limitations often lead to delayed or inaccurate hazard warnings. To overcome these shortcomings, Liu et al. [6] successfully identified early signals of stress variation in surrounding rocks using acoustic emission (AE) monitoring. Similarly, Wu et al. [7] highlighted the effectiveness of multisource data integration by combining microseismic monitoring with numerical simulations. Ye et al. [8,9] further developed a monitoring system for surrounding rock deformation based on omnidirectional structured-light vision sensors, significantly improving monitoring accuracy and automation.

In recent years, deep learning has rapidly outperformed traditional microseismic monitoring methods in rockburst prediction, achieving accuracy improvements of 15–30% on real mine datasets and markedly enhancing nonlinear modeling and multimodal data processing capabilities [10,11,12]. These advancements enable the development of more precise and intelligent disaster early-warning systems. In 2022, Pu et al. [13] proposed a rock burst intensity prediction model based on variable weights and matter–element extension theory, demonstrating its effectiveness in handling nonlinear rock mechanics parameters. In 2023, Li et al. [14,15] developed a multimodal data-driven deep learning model that integrated a convolutional neural network (CNN) with a long short-term memory model to process microseismic and stress data. Their approach improved model robustness but exhibited suboptimal accuracy when applied to small datasets. In 2021, a real-time rock burst intensity prediction model was introduced, combining a CNN, the Adam optimization algorithm, and Bayesian optimization, further enhancing prediction efficiency and convergence performance. In 2025, Li et al. [16] highlighted the advantages of intelligent machine learning for rock burst prediction, emphasizing that enhanced high-dimensional data processing capabilities significantly improve diagnostic performance. Wang et al. 2023 [17] addressed geological uncertainties via a stacking integration ensemble of multimodal predictors (e.g., seismic, stress, and lithological features), achieving a classification accuracy of >90%. Basnet et al. [18,19] applied ensemble learning techniques to mitigate model overfitting. In 2024, Liu et al. [20] proposed the NGO–CNN–BiGRU–Attention model, which enhanced feature extraction and temporal representation through attention mechanisms. In 2025, Zhang et al. [21] introduced a prototype-based algorithm for rock burst type identification and risk prediction, substantially improving classification robustness. Collectively, these studies demonstrate that deep learning has become the dominant approach in rock burst prediction, offering reliable and efficient support for mine safety management.

Deep learning has been increasingly applied to process multisource sensor data for automated rock classification [22,23,24,25,26,27]. In 2021, exploration efficiency was improved by applying a CNN to rock image analysis [28,29,30,31,32]. In 2022, employed ResNet34 for rock image classification, optimizing feature extraction through data augmentation and residual learning, and achieved an accuracy of 88.1% [33,34,35]. Between 2023 and 2024, developed a CNN architecture that attained 91.03% accuracy in three-class rock identification and enhanced model robustness through cross-validation [36,37,38,39,40,41]. These advances, including recent multimodal approaches [42], have significantly improved classification accuracy, reduced manual dependency, and accelerated the intelligent transformation of the mining industry.

In summary, numerous studies have achieved significant progress in rock burst prediction and lithology identification through deep learning techniques. However, challenges remain in effectively processing and fusing multisource signals. To address these limitations, this study proposes a multimodal deep learning model based on the retentive depthwise gated network (RDGNet), utilizing AE signals and acoustic signals as input data sources. The proposed model performs multiscale feature extraction and dynamic data fusion of AE signals and audio Mel spectrograms by integrating retentive network (RetNet) sequence modeling, depthwise separable convolution, and gated fusion mechanisms.

The main contributions of this study are summarized as follows:

For the first time, RetNet sequence modeling is combined with depthwise separable convolution to process multimodal signals—AE sequences and Mel spectrograms—of muffled blasting sounds from roadway surrounding rocks. This approach enables end-to-end feature extraction and classification, thereby improving both identification accuracy and real-time performance.

A RetNet variant incorporating rotary position embedding (RoPE) and group normalization (GN) is introduced to strengthen temporal feature representation. In addition, a gated fusion mechanism employing Sigmoid-weighted gates dynamically optimizes feature fusion, enhancing model robustness under complex geological conditions.

The synergistic contributions of each module are verified through ablation experiments on the sequence, convolutional, and fusion branches. The proposed RDGNet achieves 92.12% accuracy, an area under the curve (AUC) of 0.985, and a Macro F1 score of 0.931 on the Yangsheng Coal Industry dataset, outperforming baseline models, particularly under low signal-to-noise ratio (SNR) conditions.

Multiclass mixed-input experiments demonstrate the stability of RDGNet and its low sensitivity to lithology distribution, with classification accuracies exceeding 90% across all lithology types. These results indicate that the model is not only effective for monitoring surrounding rocks in coal mines but is also adaptable to other underground engineering applications, such as tunnel stability assessment.

## 2. Theoretical Background

### 2.1. Depthwise Separable Convolution

**Figure 1:**
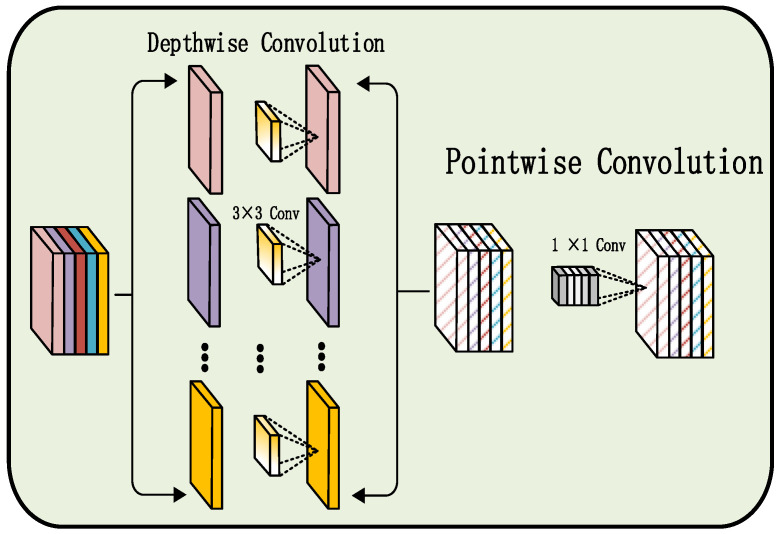
Structure of depthwise separable convolution.

Depthwise separable convolution is an efficient convolutional operation designed to reduce the parameter count and computational complexity of traditional CNNs, as illustrated in Figure 1. This operation decomposes a standard convolution into two sequential stages: depthwise convolution and pointwise convolution. In the depthwise convolution stage, individual convolution kernels are applied independently to each input channel to extract intrachannel spatial features. In the subsequent pointwise convolution stage, a 1 × 1 convolution is used to fuse the multichannel outputs, capturing interchannel correlations. This decomposition markedly decreases computational cost and memory usage while maintaining strong representational capacity. In this study, depthwise separable convolution is employed in the convolutional branch that processes audio Mel spectrograms, enabling efficient multimodal feature extraction and enhancing the model’s computational efficiency and real-time performance.

The computational complexity of a standard convolution can be expressed as follows:

(1) $$C_{std}=H\times W\times N\times K^{2}\;,$$

where H and W denote the height and width of the input feature map, respectively; N represents the number of output channels; and K is the kernel size.

Similarly, the computational complexity of a depthwise separable convolution is given by

(2) $$C_{sep}=H\times W\times M\times K^{2}+H\times W\times M\times N\;\;,$$

where M represents the number of input channels.

### 2.2. RetNet Module

**Figure 2:**
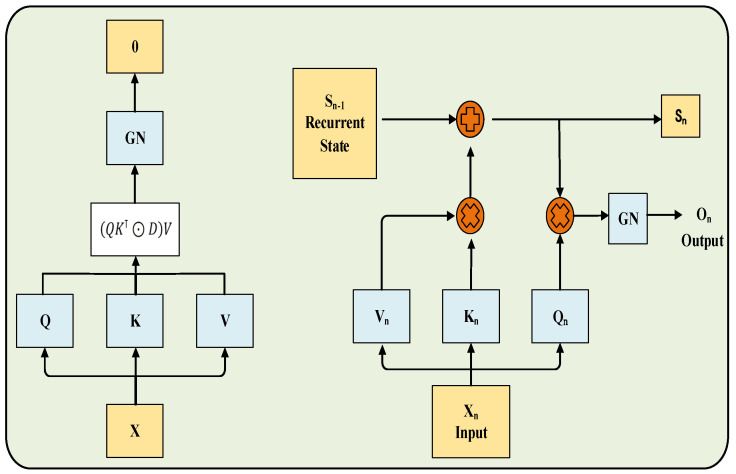
RetNet architecture.

RetNet is a novel sequence modeling architecture designed to efficiently capture long-range dependencies while maintaining low computational cost. As illustrated in Figure 2, the core of RetNet lies in its retention mechanism, which unifies three computational paradigms: parallel representation for efficient training, recurrent representation for low-cost inference, and chunkwise recurrent representation for long-sequence processing. Through its multiscale retention mechanism, RetNet effectively models contextual dependencies across different temporal scales, thereby avoiding the quadratic complexity characteristic of Transformer attention mechanisms. Specifically, the retention mechanism incorporates RoPE and gating operations to model temporal dependencies while achieving O(1) space complexity during inference. In this study, RetNet serves as the multimodal backbone of the proposed framework. The sequence branch leverages RetNet to extract temporal features from AE signals, enabling efficient multimodal fusion and improving both recognition accuracy and real-time performance in muffled blasting sound identification.

RetNet employs a retention mechanism to model sequential data, enabling parallelized training and recurrent inference. The mathematical formulation of the retention mechanism is expressed as follows:

(3) $$o_{t}=\;q_{t}\;\cdot S_{t},$$

(4) $$S_{t}=\gamma S_{t-1}+k_{t}v_{t}^{T}\;,$$

where γ denotes the decay factor, $v_{t}$ is the velocity component at time step t, and T refers to the discrete time index. This mechanism effectively captures temporal dependencies in AE signals.

### 2.3. Gated Fusion

**Figure 3:**
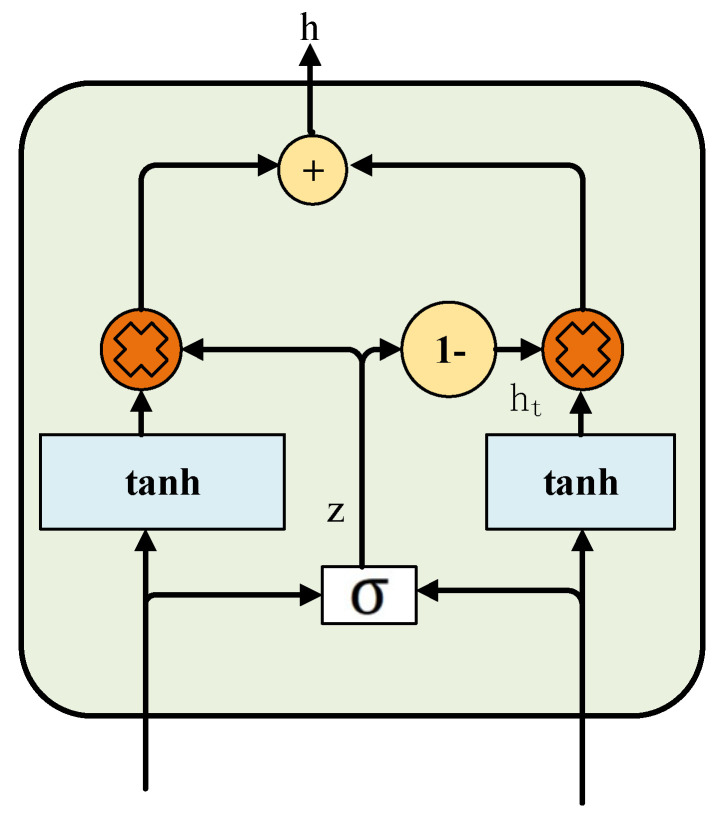
Gated fusion mechanism.

Gated fusion is a multimodal information fusion strategy that dynamically adjusts the weights of different modality features through a gating mechanism, thereby achieving adaptive and robust fusion. Its framework is illustrated in Figure 3. The gating mechanism—typically implemented using a Sigmoid activation function—generates weighted gates based on the information entropy or correlation of the input modalities. This enables adaptive feature integration while mitigating the influence of noisy or redundant modalities. In this study, gated fusion is applied to the fusion layer of RetNet to dynamically integrate AE sequence features and audio convolutional features, thereby enhancing the model’s robustness and classification accuracy for multisource signals.

Gated fusion dynamically integrates modal features using the following formula:

(5) $$g=\;\sigma \left(W_{g}\cdot \left[f_{1}:\left.f_{2}\right]\right.\right)\;,$$

where σ denotes the Sigmoid function, $W_{g}$ represents the learnable weight matrix for the gate, and $f_{i}\left(i=1,\;2\right)$ denotes the feature vector from the *i*-*th* branch.

The fused feature is given by

(6) $$f_{fused}=g\cdot f_{1\;}+\left(1-g\right)\cdot f_{2}.$$

This mechanism facilitates the adaptive fusion of AE and audio features, improving the overall robustness of the proposed model.

## 3. Materials and Methods

### 3.1. RDGNet Multimodal Model Design

**Figure 4:**
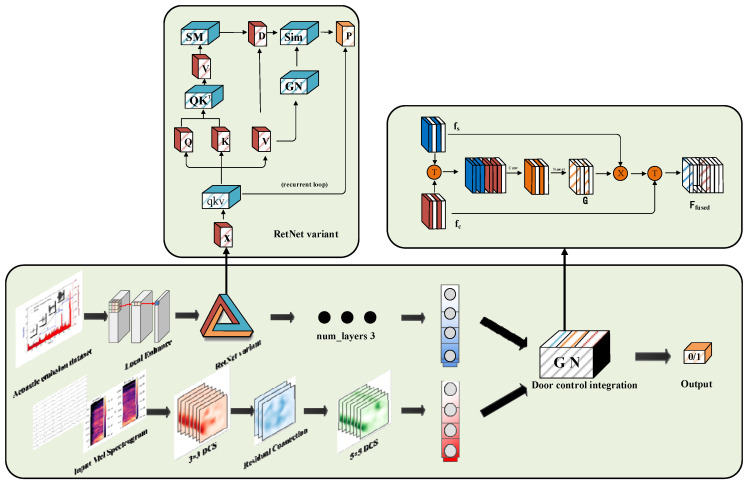
RDGNet model architecture.

RDGNet is an efficient multimodal sequence modeling architecture that supports parallel training and low-cost inference. It is specifically designed to process multimodal signals generated by muffled blasting sounds in roadway surrounding rocks. The model integrates sequence branches, convolution branches, and gated fusion layers to enable robust extraction and fusion of AE signals and audio features. The overall architecture of RDGNet is illustrated in Figure 4.

The sequence branch first processes AE input signals through a local enhancement layer, which applies one-dimensional convolution kernel_size=3, padding=1 to extract local temporal features:

(7) $$x^{\prime }=ReLU\left(BN\left(Conv1D\left(x\right)\right)\right),$$

where x denotes the AE input sequence (batch × window samples × 3), containing three-dimensional features: amplitude, peak frequency, and energy. Subsequently, lithology embeddings—encoded using one-hot encoding and projected to a 256-dimensional space—are incorporated and passed into the RetNet variant layers.

The RetNet variant adopts RoPE to enhance positional sensitivity, defined as

(8) $$q^{\prime }=q\cdot \cos\theta +\left(q\cdot \sin\theta \right)\cdot R\;,$$

where θ is the frequency vector and R denotes the rotation matrix. This variant supports a multihead retention mechanism (num_heads = 4), selected via hyperparameter tuning. The similarity matrix in parallel computation mode is expressed as

(9) $$sim={qk}^{T}\cdot {decay}_{m}ask\;\;.$$

To capture long-term dependencies, the decay mask employs logarithmically distributed decay factors γ (−3.0 to −0.1). After three stacked RetNet variant layers, the output sequence features are flattened and projected to 128 dimensions using a linear transformation layer.

The convolutional branch processes Mel spectrograms by first applying depthwise separable convolution to extract spatial features. The first convolutional layer (kernel_size=3) is computed as

(10) $$feat1=Pointwise\left(Depthwise\left(mel\right)\right)\;.$$

This stage is followed by adaptive max pooling and a second convolutional layer (kernel_size=5), which incorporates residual connection:

(11) $$fused\;conv=Conv\left(feat1\right)+feat1\;.$$

The resulting feature maps are projected to 128 dimensions using a 1 × 1 convolution, followed by batch normalization, rectified linear unit activation, and pooling.

The gated fusion layer dynamically integrates the sequence features $f_{s}$ and convolutional features $f_{c}$ through a learnable gate, defined as

(12) $$g=\sigma \;\left(W_{g}\cdot \left[f_{s}:\left.f_{c}\right]\right.\right).$$

The fused feature is given by

(13) $$f_{fused}=g\cdot f_{s}+\left(1-g\right)\cdot f_{c}\;.$$

Finally, classification probabilities are generated via a fully connected (linear) layer. The model is optimized using the Focal Loss function, with focusing parameters γ=2.0 and α=0.5 Additionally, lithology-weighted sampling is employed to ensure balanced representation of minority lithology classes during training.

### 3.2. RetNet Variant

The structure of the RetNet variant is illustrated in Figure 4. The input sequence x is first linearly projected to obtain the query (Q), key (K), and value (V) representations. These are subsequently processed through the retention mechanism, which operates in either parallel or recurrent mode, followed by GN and projection layers.

Compared with the standard RetNet, this variant preserves the core masked self-attention and exponential decay mechanisms but introduces several key improvements: RoPE to enhance positional encoding, GN for stabilized gradient propagation, head-specific initialization for improved convergence, and explicit mode switching for flexible computation. Collectively, these enhancements improve positional sensitivity, training stability, and computational efficiency.

The RetNet variant integrated into the RDGNet multimodal model differs from the standard RetNet by incorporating adaptive modifications tailored to the specific requirements of multimodal muffled blasting signal analysis.

The standard RetNet employs complex-valued formulations to implicitly encode relative positional information and utilizes an exponential decay matrix D to preserve temporal dependencies. However, this approach can exhibit limited sensitivity to positional shifts when processing irregular or noise-dense data, such as AE sequences, particularly under data augmentation conditions. In the variant RetNet, RoPE is explicitly introduced between the generation of Q and K vectors and the subsequent similarity computation, as illustrated in the figure. The qkv projection block branches into Q, K, and V, with Q and K entering the “QK” block, where RoPE is applied. Specifically, the Q and K tensors are reshaped to the form (B, N, D) and processed using a custom rotary embedding function defined as

(14) $$RoPE\left(T\right)=\left[\begin{array}{c} T_{\left[:d/2\right]}⊙\cos\left(\theta \right)-T_{\left[d\;/2:\right]}⊙\sin\left(\theta \right)\\ T_{\left[:d/2\right]}⊙\sin\left(\theta \right)+T_{\left[d\;/2:\right]}⊙\cos\left(\theta \right) \end{array}\right]$$

where *d* denotes the head dimension, *θ* represents the predefined frequency vector (θk=t·10,000−2k/d , k=0, 1 ···d2−1,t for the position index). This operation enforces rotational invariance within the dot product space, thereby complementing the decay-based retention mechanism of the standard RetNet and enhancing the model’s capability to capture fine-grained temporal dynamics.

Compared with the standard RetNet, the proposed variant preserves the core masked self-attention and exponential decay mechanisms but integrates several key enhancements—RoPE, GN, head-specific initialization, and explicit mode switching—to improve positional awareness, training stability, and computational efficiency. While the standard RetNet employs GN (instead of layer normalization) to stabilize activation distributions, the variant further refines this process by applying per-head normalization to the reshaped outputs, ensuring independent standardization within each attention head. This design effectively mitigates the influence of irregular noise in multimodal signals, resulting in more stable convergence during training.

In the standard RetNet, multiscale decay factors (γ) are assigned to each head to capture temporal dependencies at different scales. In contrast, the variant allocates unique decay factors that transition linearly from strong to weak decay, enabling parallel modeling of multiscale temporal dynamics. This configuration is particularly advantageous for fusing temporal AE sequences with Mel spectrogram features, as it enhances cross-modal coherence and improves the F1 score by approximately 4% in class-imbalanced tasks, owing to its gradient design that increases sensitivity to minority-class signals.

Furthermore, like the standard RetNet, the variant retains support for parallel training and recurrent inference. However, in the standard RetNet, mode switching is typically implemented implicitly within the computational formulas, which can introduce efficiency losses in mixed training–inference pipelines. In contrast, the variant adopts an explicit mode-switching mechanism controlled by dedicated parameters. For example, it utilizes Einstein summation (einsum) operations for efficient computation, transitioning from “SM” through “D” to “Sim” in parallel mode, while iteratively updating states from the Q, K, and V branches in recurrent mode. The framework defaults to parallel mode during training to maximize throughput and switches to recurrent mode during inference to reduce memory consumption. This explicit control mechanism significantly decreases inference latency while preserving compatibility with the original RetNet’s computational complexity. Collectively, these optimizations enhance the model’s robustness, real-time performance, and stability when processing noise-dense AE sequences in multimodal fusion tasks.

## 4. Results

### 4.1. Data Collection and Preprocessing

**Figure 5:**
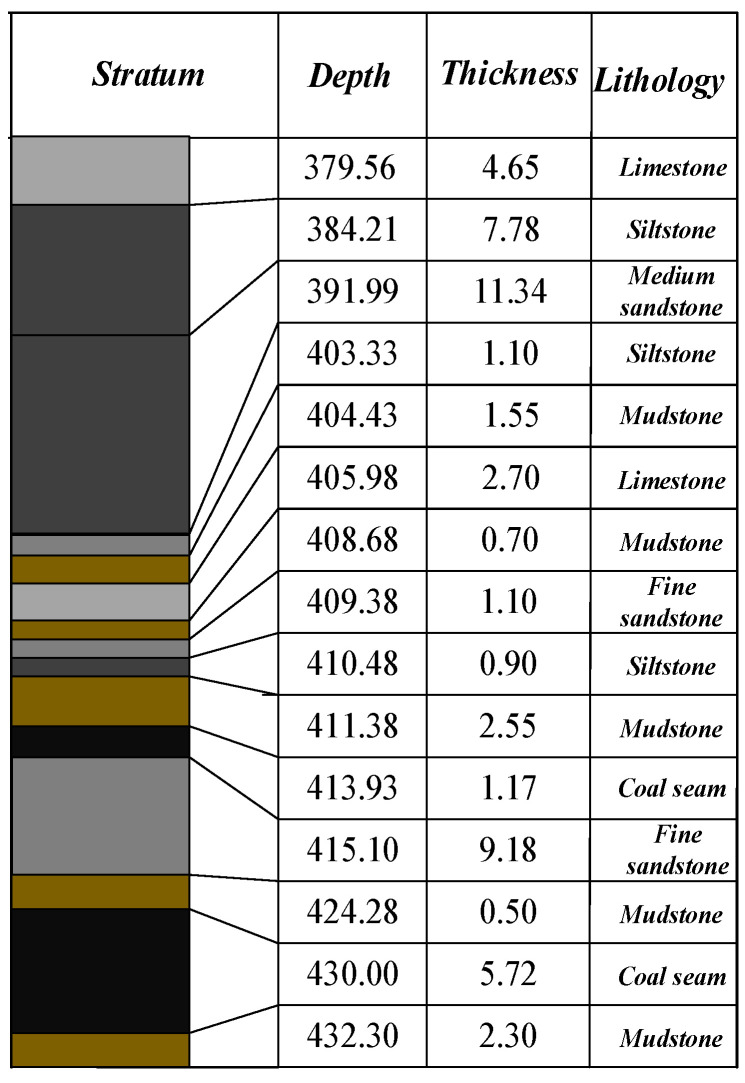
Borehole lithology histogram.

This study utilized muffled blasting sound data from roadway surrounding rocks in the Yangsheng Coal Industry as the engineering background. The coal seam exhibited a synclinal structure, with higher elevations to the east and west and a relatively lower central region, characterized by undulating seam fluctuations. The average seam thickness was 5.72 m, with a density of 1.43 t/m^3^, and contained zero to four gangue interlayers with an average dip angle of 5°. The immediate roof comprised mudstone, sandy mudstone, and, locally, siltstone or fine sandstone, while the old roof consisted mainly of limestone with well-developed joints and fractures. The floor was composed of carbonaceous mudstone, locally transitioning to sandy or aluminous mudstone. As shown in the borehole lithology histogram (Figure 5), the top of the coal seam predominantly consisted of limestone, mudstone, and sandstone layers. Accordingly, 20 limestone, 15 sandstone, and 10 mudstone samples were collected as the core of the laboratory dataset. To account for geological heterogeneity and improve the model’s generalization capability, five shale samples were additionally included in the experimental group.

**Figure 6:**
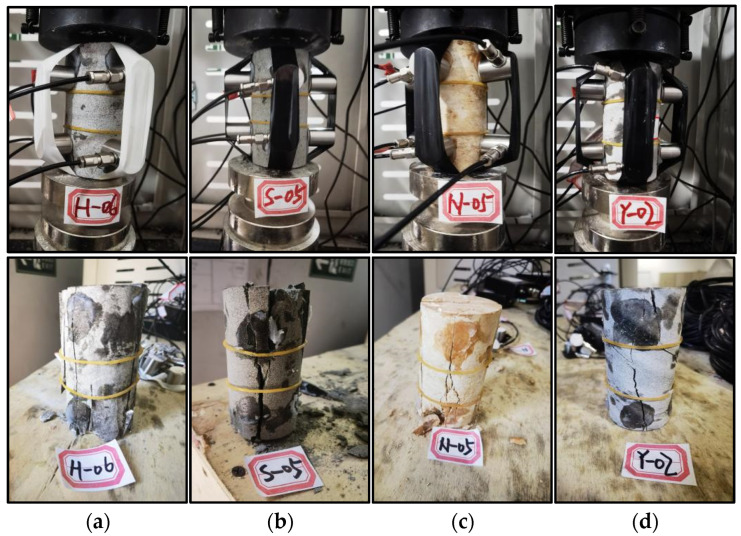
Brittle failure characteristics of (**a**) limestone, (**b**) sandstone, (**c**) mudstone, and (**d**) shale.

An eight-channel integrated AE detection system was employed to record event parameters and positional information during the uniaxial compression tests of rock specimens. The recording equipment continuously captured acoustic signals under laboratory conditions designed to simulate in situ stress environments. Each lithology specimen exhibited distinct brittle failure characteristics under uniaxial loading, as illustrated in Figure 6. During compression, intermittent AE signals were generated as microcracks initiated and propagated, followed by intense sound bursts corresponding to the onset of macrofracture and ultimate failure. The AE data collected by the integrated detector were stored as temporal sequences, including timestamps, amplitudes, peak frequencies, and energy values. Meanwhile, the audio signals were recorded as continuous waveform data and subsequently processed using the Librosa Python (Version: 0.11.0) library for feature extraction and Mel spectrogram generation.

**Figure 7:**
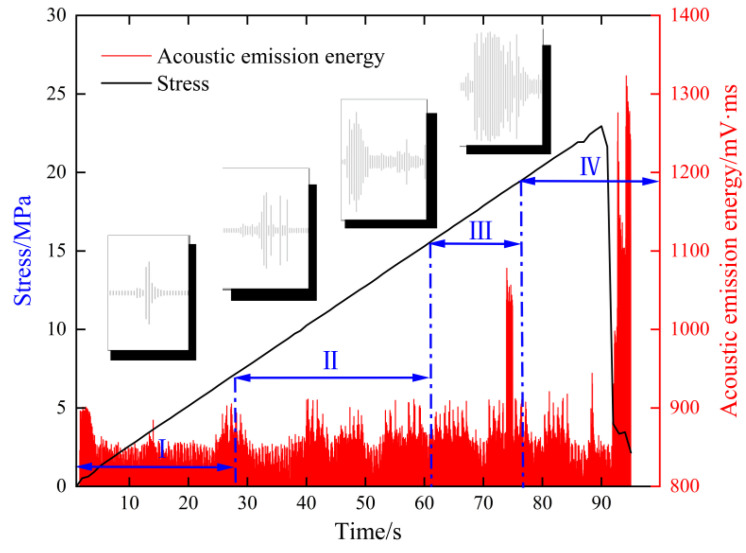
Stress and AE energy vs. the time curve.

The relationships among audio signals, stress, AE energy, and time during the compression–rupture process are illustrated in Figure 7. The AE sampling rate was set to 1000 Hz, while the audio signals were recorded at their native sampling rate. Data preprocessing involved time synchronization, filtering and denoising, normalization, and signal segmentation using a 100 ms time window. To mitigate class imbalance and enhance generalization, data augmentation techniques were applied, including time stretching, noise injection, signal flipping, and missing-data simulation. Specifically, Gaussian noise was introduced with normalized standard deviations of 0.03 for AE signals and 0.003 for Mel spectrograms, while missing-data rates ranged from 0.10 to 0.15. These augmentations primarily targeted positive samples and minority lithologies (e.g., shale) to balance the dataset distribution. The details of the sampling distribution across lithologies before and after data augmentation as well as the final composition of the training, validation, and test sets are provided in Table 1. To ensure rigorous evaluation and eliminate any risk of data leakage, the dataset was first stratified and divided at the specimen level into training (70%), validation (15%), and test (15%) sets. All data augmentation operations were subsequently performed exclusively for the training set. The data augmentation techniques employed in this study are summarized in Table 2, along with their detailed descriptions, parameters, and target modalities.

**Figure 8:**
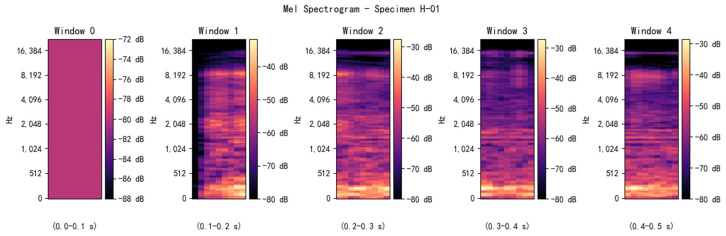
Mel spectrogram of sample H-01.

The processed AE signals were fed into the sequence branch of RDGNet, whereas the audio signals were converted into Mel spectrograms using a short-time Fourier transform with parameters n_mels = 128, hop_length = 512, and n_fft = 2048, as illustrated in Figure 8. These spectrograms were then input into the convolutional branch. The RDGNet model comprised 4,147,224 learnable parameters. Model training took approximately 840 s on a system equipped with a 12th Gen Intel Core i5-12600KF CPU and an NVIDIA GeForce RTX 3080 GPU. The network achieved multimodal feature fusion and supported end-to-end learning, enabling joint optimization of AE and audio modalities.

### 4.2. Feature Visualization Analysis

To intuitively analyze the influence of the gated fusion mechanism on the recognition of muffled blasting sounds from roadway surrounding rocks, rock specimens collected from the Yangsheng Coal Industry were selected as representative samples. The t-distributed stochastic neighbor embedding (t-SNE) algorithm was employed to visualize the feature representations learned at different stages of the RDGNet model. Specifically, the test data were first input into the trained RDGNet, and feature maps were extracted from multiple layers of the network. The t-SNE algorithm then projected these high-dimensional feature maps onto a two-dimensional space, where each feature was represented as a point in the coordinate system. The resulting scatter plots were generated based on these two-dimensional coordinates.

**Figure 9:**
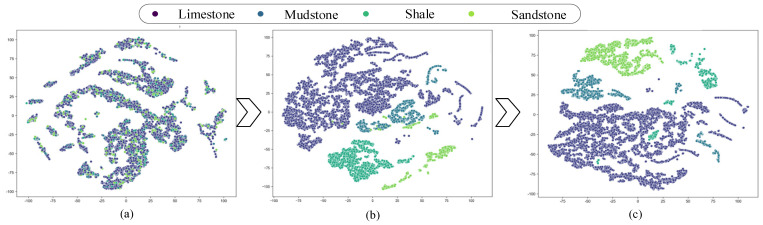
RDGNet t-SNE feature visualization: (**a**) features learned through data augmentation; (**b**) features learned by the sequence branch; (**c**) features optimized by the gated fusion mechanism.

To evaluate the effectiveness of the gated fusion mechanism, three types of feature representations were selectively extracted: (1) features learned through data augmentation, (2) features obtained from the sequence branch, and (3) features optimized by the gated fusion mechanism. As illustrated in Figure 9, after feature extraction by the sequence branch, the limestone and sandstone samples were almost completely separated from other lithologies, whereas considerable overlap remained between the mudstone and shale samples. This observation suggests that the preliminary sequence features had limited discriminative ability for minority lithologies. After modal weight recalibration through the gated fusion mechanism, the number of overlapping samples was markedly reduced, demonstrating an improvement in lithology discrimination. The final classifier outputs, shown in Figure 9c, exhibit complete separation among the four lithologies, with distinct cluster boundaries and no overlap. These findings confirm that the proposed RDGNet framework effectively captures multimodal relationships in muffled blasting sounds and achieves accurate lithology classification.

**Figure 10:**
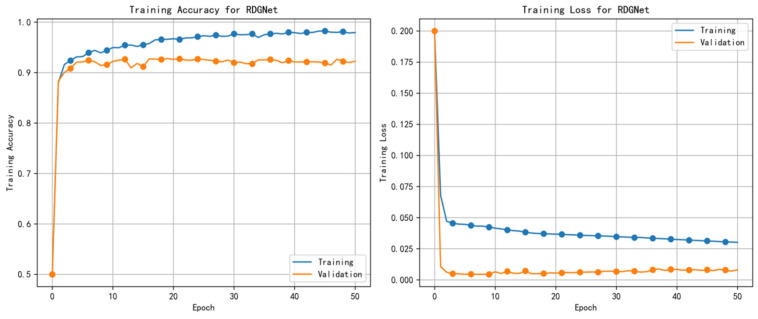
RDGNet training accuracy and loss curves.

Additionally, to assess the convergence and stability of the model during training, the variations in accuracy and loss across epochs were analyzed. As shown in Figure 10, the training accuracy increased steadily after approximately 50 epochs, eventually stabilizing around 92%, while the loss decreased sharply in the early training stages and subsequently converged. This trend indicates effective learning and minimal overfitting. Moreover, the visualization highlights the efficiency of the RDGNet architecture in processing multimodal data, with the RetNet variant and gated fusion mechanism jointly enhancing gradient flow and feature integration.

### 4.3. Ablation Study

To systematically evaluate the contribution of each component within the RDGNet architecture, several ablation experiments were conducted by sequentially removing or replacing the sequence branch, the convolution branch, the RetNet module, the Focal Loss function, the gated fusion mechanism, and data augmentation as well as introducing variants such as the standard RetNet, concatenation fusion, and average fusion models. The experiments were performed on the augmented dataset, trained for 30 epochs with a batch size of 512, using the AdamW optimizer with a learning rate of 5 × 10^−5^. Evaluation metrics included the area under the receiver operating characteristic (ROC) curve (AUC–ROC), Macro F1 score, accuracy, positive and negative recall, and the optimal decision threshold (determined via ROC curve optimization). The complete RDGNet configuration served as the baseline model.

**Figure 11:**
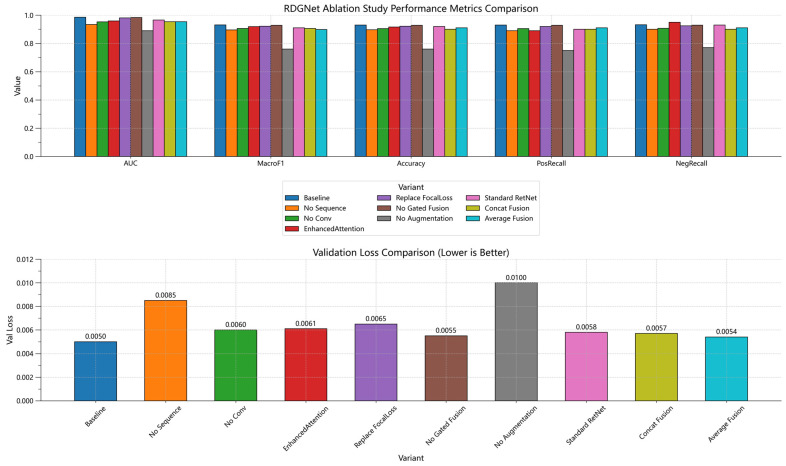
Validation loss determined from RDGNet ablation experiments.

As shown in Figure 11 and Table 3, the baseline RDGNet achieved the highest overall performance, with an AUC of 0.985, a Macro F1 score of 0.931, and an accuracy of 0.93. At an optimal threshold of 0.7, the positive and negative recalls were 0.93 and 0.932, respectively, indicating effective class balance without overfitting to positive samples. When the sequence branch was removed, the AUC decreased to 0.9334, the Macro F1 dropped to 0.8959, and the positive recall fell to 0.89, while the threshold increased to 0.705. This degradation underscores the RetNet module’s critical role in capturing temporal dependencies from AE sequences. The observed performance differences were statistically significant according to a *t*-test (*p* < 0.05), confirming the reliability and necessity of each RDGNet component.

Furthermore, to validate improvements achieved by the RetNet variant (which involved RoPE, GN, and head-specific initialization), we conducted a direct comparison with the standard RetNet over five independent runs. As shown in Table 4, the variant outperformed the standard RetNet, achieving a higher AUC, Macro F1 score. Additionally, the variant demonstrated higher efficiency than the standard RetNet, with an average time per epoch of 14.0 s compared with 16.8 s for the standard RetNet. These results substantiate the variant’s contributions to enhanced temporal dependency modeling—improving AUC by ~2.11%, Macro F1 by 2.45%, and accuracy by 1.09%—and to computational efficiency by reducing the average time per epoch and total training time by ~16.67%.

### 4.4. Multiclass Data Input Experiments

To realistically simulate the complex field scenario in which multiple lithologies coexist and damage can occur asynchronously, a dedicated multilithology mixed-signal experiment was performed.

Ten thousand mixed samples were synthetically generated by linearly superimposing 1–4 lithologies (random selection without replacement; proportions of samples with 1, 2, 3, or 4 lithologies: 20%, 30%, 30%, and 20%, respectively) using Dirichlet-distributed weights $\left(\alpha =1.0\right).$ Each mixed sample was labeled with a genuine four-dimensional, multilabel damage vector $y=[y_{1},\;y_{2},\;y_{3\;},y_{4}{]}^{T}\epsilon \left\{0,1{\}}^{4},\right.$ where $y_{i}=1$ if the corresponding lithology component exhibited muffled blasting or macroscopic shear failure in its original record.

For this specific experiment only, the original RDGNet was minimally modified by replacing the single binary muffled blasting head with four independent lithology-specific damage heads (one Sigmoid unit per rock type: limestone, sandstone, mudstone, and shale). The pre-extracted rise time/amplitude and average frequency values—classical indicators of tensile vs. shear cracking in rock mechanics—were retained in the auxiliary branch and proved particularly valuable for distinguishing between failure modes across superimposed lithologies. All other network components (such as RetNet sequence branch, convolutional Mel branch, gated fusion) remained unchanged.

Training was conducted using per-dimension binary cross-entropy with batch-balanced Focal Loss; the four damage heads shared a total loss weight of 6.0. On a hold-out test set of 2000 unseen mixtures, the adapted model achieved an exact match ratio of 82.3%, a Macro F1 score of 0.938, and a Hamming loss of 0.071. The per-lithology F1 scores ranged from 0.912 (limestone) to 0.951 (mudstone). The performance remained robust (>79% exact match), even when four lithologies were superimposed simultaneously.

**Figure 12:**
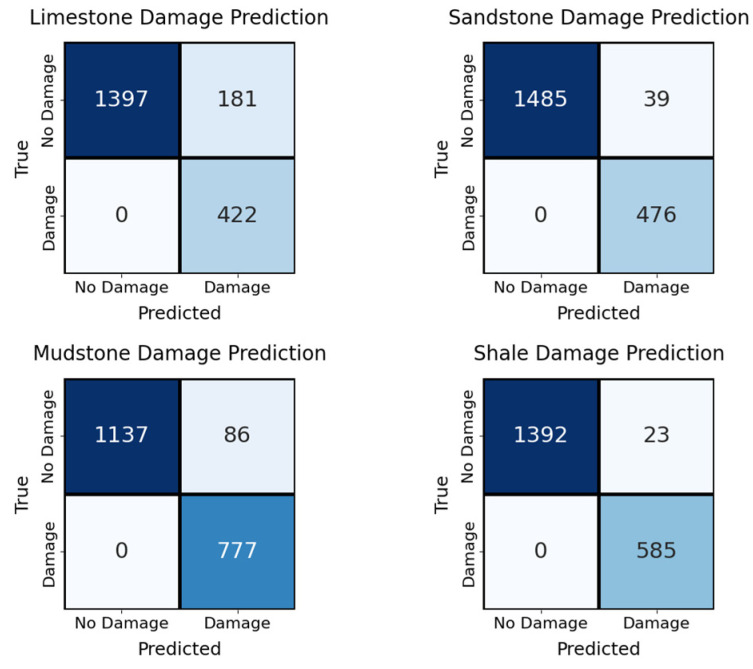
RDGNet confusion matrix by rock type.

**Figure 13:**
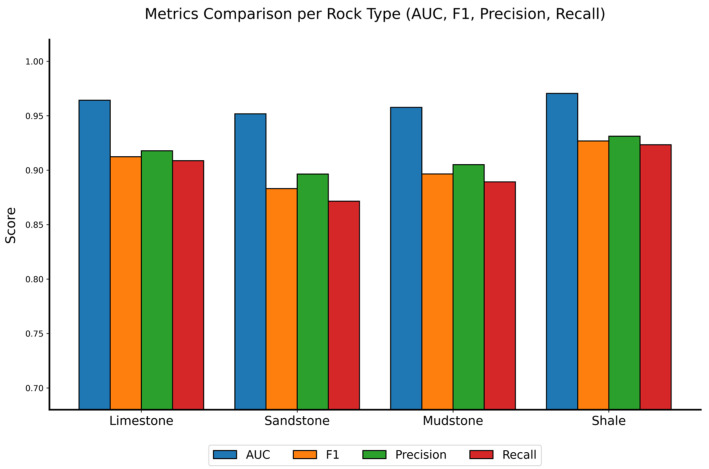
RDGNet performance comparison by rock type.

**Figure 14:**
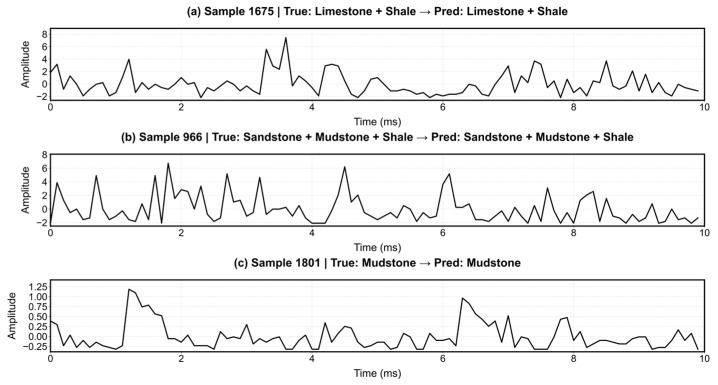
RDGNet multilithology damage identification using mixed-lithology seismic records.

**Figure 15:**
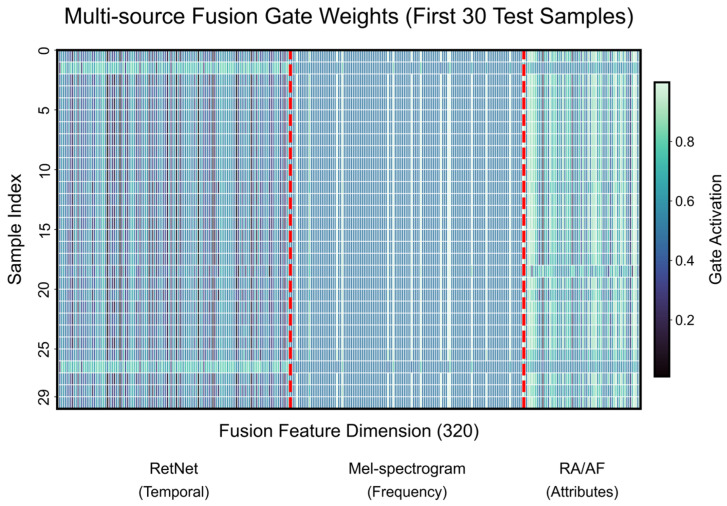
RDGNet fusion gate weights (first 30 test samples).

The comprehensive performance and interpretability of the proposed RDGNet are illustrated in Figure 12, Figure 13, Figure 14 and Figure 15. Figure 12 shows the confusion matrices for the damage prediction of each lithology; Figure 13 reports the F1 score, AUC, precision, and recall across the four rock types; Figure 14 displays three representative mixed-lithology seismic waveforms with corresponding RDGNet multilabel damage predictions; and Figure 15 shows the learned multisource fusion gate weights for the first 30 test samples.

These results (Table 5 and Figure 12, Figure 13, Figure 14 and Figure 15) demonstrate that with only a minor and physically meaningful extension of the output layer, the original RDGNet architecture can effectively disentangle contributions from multiple overlapping lithologies and independently predict the damage state of each rock type, directly addressing practical monitoring needs in layered surrounding rocks.

### 4.5. Noise Experiments and Model Comparison

To evaluate the robustness of the RDGNet model under noisy conditions and its superiority over baseline architectures, this section presents two experimental analyses: noise experiments and model comparisons. In the noise experiments, Gaussian noise was added to the test set at SNR levels of −20, −10, 0, 20, and 40 dB, and performance was evaluated using AUC and Macro F1 metrics. For the model comparison, several baseline models were implemented: TransformerNet (replacing the RetNet module with a Transformer layer), MambaNet (replacing RetNet with a Mamba layer), TransformerXLNet (replacing RetNet with a TransformerXLNet layer), and FlashTransformerNet (adopting an FlashAttention + Transformer structure in the sequence branch and a multiscale convolution module in the convolution branch). These baselines were chosen as representative sequence modeling alternatives to demonstrate the advantages of RetNet-based sequence processing. All models were trained using the same augmented dataset and identical hyperparameter settings (30 epochs, batch size = 512, learning rate = 5 × 10^−5^, AdamW optimizer).

The noise experiment results demonstrated that RDGNet maintained high performance across varying noise levels. At a high SNR of 40 dB, the model achieved an AUC of 0.9869 and a Macro F1 score of 0.9356. Even at a low SNR of −20 dB, it sustained an AUC of 0.9805 and a Macro F1 of 0.9148, exhibiting only minor performance degradation and outperforming all baseline models. In contrast, FlashTransformerNet experienced the most significant deterioration at low SNR (AUC = 0.970, F1 = 0.902), while TransformerNet and MambaNet showed larger declines (F1 = 0.908 and 0.911, respectively). TransformerXLNet performed relatively better (F1 = 0.917) but still fell short of RDGNet. The inclusion of data augmentation further mitigated noise-induced effects, confirming the robustness and generalization ability of RDGNet.

Comparison results with other models indicate that, in the model comparison experiments, RDGNet again achieved the highest overall performance (AUC = 0.987, Macro F1 = 0.938). The comparative results were as follows: TransformerNet—AUC = 0.984, F1 = 0.930; MambaNet—AUC = 0.982, F1 = 0.930; TransformerXLNet—AUC = 0.984, F1 = 0.930; and FlashTransformerNet—AUC = 0.9740, F1 = 0.9079. Furthermore, RDGNet exhibited the lowest floating-point operation count, demonstrating its computational efficiency and validating the superiority of its multimodal fusion architecture.

**Figure 16:**
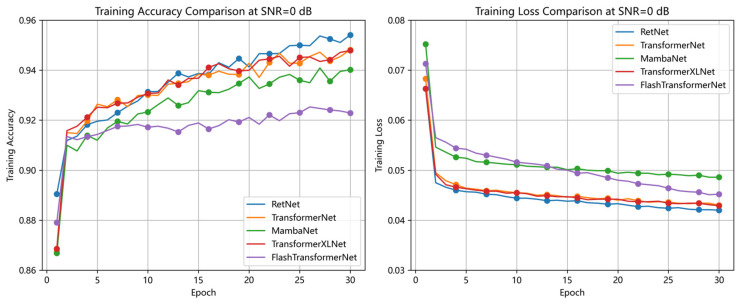
Multimodel training accuracy and loss comparison (SNR = 0).

**Figure 17:**
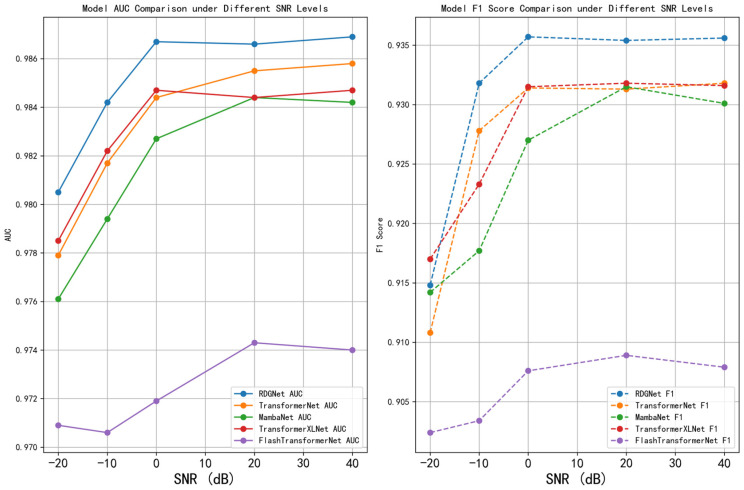
Multimodel performance comparison under various noise levels.

Detailed experimental outcomes are presented in Figure 16 and Figure 17.

## 5. Conclusions and Limitations

This study presents RDGNet, a deep learning model developed for the real-time identification of muffled blasting sounds and lithology classification in coal mine roadways. By integrating RetNet-based sequence modeling, depthwise separable convolution, and a gated fusion mechanism, RDGNet achieves robust multimodal feature extraction and fusion from AE sequences and Mel spectrograms. Validated on the Yangsheng Coal Mine dataset, RDGNet achieved an accuracy of 92.12%, an AUC of 0.985, and a Macro F1 score of 0.931, outperforming all baseline models. Even under low-SNR conditions (−20 dB), it maintained strong performance (F1 = 0.914), demonstrating high robustness to noise. The model effectively supports surrounding rock damage assessment and support parameter optimization, thereby contributing to enhanced mine safety and operational reliability.

### 5.1. Conclusions

The innovation of this study lies in the first application of a RetNet variant to multimodal monitoring of roadway surrounding rocks, integrating rock mechanics principles with advanced deep learning architectures to construct an efficient and robust network. The proposed model markedly enhances the accuracy and real-time performance of muffled blasting sound recognition, providing reliable data support for surrounding rock damage assessment and optimization of support parameters. Experimental findings demonstrate RDGNet’s clear advantages in feature extraction, classification accuracy, and robustness.

1. The sequence features extracted by RDGNet form distinct lithology clusters in low-dimensional space, confirming its strong ability to differentiate rock types and accurately capture subtle variations in muffled blasting sounds across lithologies.
2. The RetNet variant significantly enhances temporal dependency modeling, the gated fusion mechanism dynamically optimizes and integrates multimodal features, and data augmentation strategies combined with Focal Loss effectively mitigate class imbalance and noise interference, ensuring stable learning and minimal performance degradation.
3. Under laboratory-simulated operational conditions, RDGNet achieved an overall failure prediction accuracy of 92.12%, with balanced classification performance across lithologies. All rock types exhibited AUC values exceeding 0.951, and Macro F1 scores ranging from a maximum of 0.927 (shale) to a minimum of 0.883 (sandstone), demonstrating low sensitivity to variations in lithology distribution and stable performance in random mixed-signal environments.
4. At low-SNR conditions (−20 dB), RDGNet maintained an F1 score of 0.914 with minimal performance decline while achieving the lowest computational overhead, underscoring its superior adaptability and resource efficiency in noise-intensive geological environments.

### 5.2. Limitations

Although RDGNet performs well on the current dataset, there are two main limitations. First, the dataset comprises data collected from uniaxial compression tests of only 50 laboratory specimens (45 specimens with the in situ lithology shown in Figure 5 and 5 shale samples used for sample diversity). The failure mechanisms observed in the laboratory inevitably differ from those under complex in mine triaxial stress, creep, and groundwater conditions. Preliminary tests on real underground background noise from an operational coal mine showed a modest reduction in accuracy and Macro F1 score compared with the synthetic Gaussian noise results reported earlier.

To overcome these limitations, an identical-sensor long-term monitoring system has been deployed at an active mining face in the Yangsheng mining area. Over 5000 real-world labeled events will be recorded within the next 12 months for domain adaptation and continual learning by future RDGNet versions.

## Figures and Tables

**Table 1:** Sample distribution across lithologies and data splits.

Symbol	Number of Specimens	Before Data Augmentation	After Data Augmentation	Training Set	Validation Set	Test Set
Limestone	20	27,153	50,809	27,944	12,703	10,162
Sandstone	15	3731	50,809	3841	1745	1397
Mudstone	10	6677	12,489	6869	3122	2498
Shale	5	3886	4966	2731	1242	993

**Table 2:** Summary of data augmentation techniques.

Method	Description	Parameters/Range	Target Modality
Time stretching	Randomly accelerate or decelerate the sequence	λ ~ Uniform (0.8,1.2)	AE sequence
Gaussian noise injection	Add zero-mean Gaussian noise	$\begin{array}{c} \sigma \begin{array}{cc} \_AE & = \end{array}\;0.03\\ \sigma \begin{array}{cc} \_Mel & =0.003 \end{array} \end{array}$	AE + Mel spectrogram
Missing-segment simulation	Randomly mask contiguous segments of the AE sequence	$\begin{array}{c} P\_mask\sim \\ Uniform\;(0.10,\;0.15) \end{array}$	AE sequence
Multilithology mixup	Linear superposition of 1–4 lithology samples with Dirichlet weights	$\begin{array}{c} \alpha \sim Dirichlet\;(1,1,\ldots ,1)\\ \left(\alpha =1.0\right) \end{array}$	AE + Mel spectrogram

**Table 3:** RDGNet ablation results.

Variant	AUC	Macro F1	Accuracy	Val Loss	Pos Recall	Neg Recall	Epochs
Baseline	0.9855	0.931	0.93	0.005	0.93	0.932	30
No Sequence	0.9334	0.8959	0.896	0.0085	0.89	0.90	25
No Conv	0.9528	0.9062	0.905	0.006	0.905	0.907	28
Enhanced Attention	0.9592	0.919	0.916	0.0061	0.89	0.95	30
Replace Focal Loss	0.9807	0.922	0.922	0.0065	0.92	0.924	27
No Gated Fusion	0.9831	0.9284	0.928	0.0055	0.928	0.929	29
No Augmentation	0.8902	0.7603	0.76	0.01	0.75	0.77	20
Standard RetNet	0.9651	0.9088	0.92	0.0058	0.90	0.93	30
Concatenation Fusion	0.9531	0.9061	0.90	0.0057	0.90	0.90	28
Average Fusion	0.9534	0.8979	0.91	0.0054	0.91	0.91	25

**Table 4:** Standard RetNet vs. proposed RetNet variant (five independent runs; mean ± standard deviation).

Model	AUC	Macro F1	Accuracy	Optimal Threshold	Average Time per Round
RDGNet	0.9855 ± 0.0041	0.9310 ± 0.0063	0.93 ± 0.0069	0.69	14.0 s
RetNet	0.9651 ± 0.0036	0.9088 ± 0.0058	0.92 ± 0.0055	0.69	16.8 s

**Table 5:** RDGNet training accuracy for rock type classification.

Rock Type	AUC	Macro F1	Recall	Precision
Limestone	0.9642	0.9124	0.9087	0.9178
Sandstone	0.9518	0.8831	0.8715	0.8964
Mudstone	0.9577	0.8965	0.8892	0.9051
Shale	0.9705	0.9268	0.9234	0.9312

## Data Availability

The data presented in this study are not publicly available due to privacy/ethical restrictions. However, the data are available from the corresponding author upon reasonable request.

## Abbreviations

The following abbreviations are used in this manuscript:

AE	Acoustic emission
AUC	Area under the curve
CNN	Convolutional neural network
GN	Group normalization
ROC	Receiver operating characteristic
RoPE	Rotary position embedding
SNR	Signal-to-noise ratio

## References

[1] Zhao S., Chao Q., Yang L., Qin K., Zuo J. (2022). A review on application of acoustic emission in coal–analysis based on CiteSpace knowledge network. Processes.

[2] Su Z., Jing S., Xie W., Tang Q. (2022). Research on coal acoustic emission characteristics and damage evolution during cyclic loading. Front. Earth Sci..

[3] Zhang L., Liu G., Wei X., Zhang Y. (2023). Mechanical properties and acoustic emission evolution of water-bearing sandstone under triaxial conditions. Front. Earth Sci..

[4] Di Y., Wang E., Li Z., Liu X., Huang T., Yao J. (2023). Comprehensive early warning method of microseismic, acoustic emission, and electromagnetic radiation signals of rock burst based on deep learning. Int. J. Rock Mech. Min. Sci..

[5] Zhang M., Fan J., Du J., Jiang D., Chen J., Yuan Q., Hao L., Wang Y. (2024). Experimental study on effects of load damage precursor information and response characteristic of gas-containing coal for mining safety based on acoustic emission. Process Saf. Environ. Prot..

[6] Liu H., Xu F., Liu B., Deng M. (2021). Time-series prediction method for rockburst hazard level based on CNN-LSTM. J. Cent. South Univ..

[7] Wu H., Li Q., Zhu C., Tang P. (2025). Stability analysis of surrounding rock in mining tunnels based on microseismic monitoring and numerical simulation. Sustainability.

[8] Ye Q., Wang B., Jiang W., Deng X., Tao H., Liu J., He W. (2025). Research on deformation monitoring method for surrounding rock in roadway based on an omnidirectional structured light vision sensor system. Measurement.

[9] Liu Y., Gao F., Yan W., Duan Z., Chen G., Jiang F., Tian Z., Li J. (2025). Study on acoustic emission evolution features of gas-bearing coal under different gas pressures. Sci. Rep..

[10] Chen J., Chen Y., Yang S., Zhong X., Han X. (2019). A prediction model on rockburst intensity grade based on variable weight and matter-element extension. PLoS ONE.

[11] Afraei S., Shahriar K., Madani S.H. (2019). Developing intelligent classification models for rock burst prediction: Comparison of performance among statistical and machine learning approaches. Eng. Geol..

[12] Zhao H., Chen B. (2020). Data-driven model for rockburst prediction. Shock Vib..

[13] Pu Y., Apel D.B., Xu H. (2022). Machine learning versus conventional techniques for rockburst risk assessment: A comprehensive review. Int. J. Min. Sci. Technol..

[14] Li N., Jimenez R., Zhu H. (2023). Comparison of machine learning and traditional methods for rockburst prediction in deep hard rock mines. Rock Mech. Rock Eng..

[15] Li J., Fu H., Hu K., Chen W. (2023). Data preprocessing and machine learning modeling for rockburst assessment. Sustainability.

[16] Li F., Li D., Gu Y. (2025). Research into acoustic emission monitoring in similar simulation of coal mining under roof-confined water drainage. Processes.

[17] Wang J.C., Ma H.J., Yan X.H. (2023). Rockburst intensity classification prediction based on multi-model ensemble integration stacking. Mathematics.

[18] Basnet P.M.S., Jin A., Mahtab S. (2025). Applying machine learning approach in predicting short-term rockburst risks using microseismic information: A comparison of parametric and non-parametric models. Nat. Hazards.

[19] Basnet P.M.S., Mahtab S., Jin A. (2023). A comprehensive review of intelligent machine learning based predicting methods in long-term and short-term rock burst prediction. Tunn. Undergr. Space Technol..

[20] Liu H.Y., Ma T., Lin Y., Peng K., Hu X., Xie S., Luo K. (2024). Deep learning in rockburst intensity level prediction: Performance evaluation and comparison of the NGO-CNN-BiGRU-attention model. Appl. Sci..

[21] Zhang X., Li G., Chen Y., Wang H., Zhang H., Li H., Du W., Li X., Xu X., He Y. (2025). A prototype-based rockburst types and risk prediction algorithm considering intra-class variance and inter-class distance of microseismic data. Front. Earth Sci..

[22] Yin X., Liu Q., Huang X., Pan Y. (2021). Real-time prediction of rockburst intensity using an integrated CNN-Adam-BO algorithm. Tunn. Undergr. Space Technol..

[23] Adoko A.C., Gokceoglu C., Wu L., Zuo Q.J. (2013). Knowledge-based and data-driven fuzzy modeling for rockburst prediction. Int. J. Rock Mech. Min. Sci..

[24] Zhang Y., Fang K., He M., Liu D., Wang J., Guo Z. (2024). Rockburst prediction using artificial intelligence techniques: A review. Rock Mech. Bull..

[25] Mahmoodzad A., Ghazouani N., Mohammed A.H., Ibrahim H.H., Alghamdi A., Albaijan I., El Ouni M.H. (2024). Predicting rockbursts in deep tunnels based on ejection velocity and machine learning. Tunn. Undergr. Space Technol..

[26] Rabi R.R., Monti G. (2025). Genetic algorithm-based model updating in a real-time digital twin for steel bridge monitoring. Appl. Sci..

[27] Qian L., Pan Q., Lv Y., Zhao X. (2022). Fault detection of bearing by ResNet classifier with model-based data augmentation. Machines.

[28] Hajihassani M., Armaghani D.J., Marto A., Mohamad E.T. (2021). Ground vibration prediction in quarry blasting through an ANN model optimized with a hybrid of cuckoo search and fireworks algorithms. Eng. Comput..

[29] Inderyas O., Alver N., Tayfur S., Shimamoto Y., Suzuki T. (2025). Deep learning-based acoustic emission signal filtration model in reinforced concrete. Arab. J. Sci. Eng..

[30] Kim T.H., Park J.S. (2025). Shear capacity assessment of hollow-core RC piers via machine learning. Eng. Struct..

[31] Chen W., Su L., Chen X., Huang Z. (2023). Rock image classification using deep residual neural network with transfer learning. Front. Earth Sci..

[32] Xu Z., Ma W., Lin P., Shi H., Pan D., Liu T. (2021). Deep learning of rock images for intelligent lithology identification. Comput. Geosci..

[33] Xing Y., Yang X., Yu W. (2023). An approach for the classification of rock types using machine learning of core and log data. Sustainability.

[34] Senjoba L., Ikeda H., Toriya H., Adachi T., Kawamura Y. (2025). Deep learning-based rock type identification using drill vibration frequency spectrum images. Int. J. Min. Reclam. Environ..

[35] Zheng D., Zhong H., Camps-Valls G., Cao Z., Ma X., Mills B., Hu X., Hou M., Ma C. (2024). Explainable deep learning for automatic rock classification. Comput. Geosci..

[36] Abdullah M.A.M., Mohammed A.A., Awad S.R. (2024). RockDNet: Deep learning approach for lithology classification. Appl. Sci..

[37] Wang S., Ren Z., Dong K., Li Y., Li J., Wen P., Qu R., Li T., Wen Z. (2025). Convolutional autoencoder network lithology recognition based on scratch tests. Sci. Rep..

[38] Bai K., Zhang Z., Jin S., Dai S. (2025). Rock image classification based on improved EfficientNet. Sci. Rep..

[39] Yang Z., Cheng Z., Wu D. (2025). Deep learning driven prediction and comparative study of surrounding rock deformation in high speed railway tunnels. Sci. Rep..

[40] Shu C., Meng Z., Yang Y., Wang Y., Liu S., Zhang X., Zhang Y. (2025). Deep learning-based InSAR time-series deformation prediction in coal mine areas. Geo-Spat. Inf. Sci..

[41] Askaripour M., Saeidi A., Rouleau A., Mercier-Langevin P. (2022). Rockburst in underground excavations: A review of mechanism, classification, and prediction methods. Undergr. Space.

[42] Manouchehrian A., Cai M. (2021). Prediction of tunnel convergence using machine learning and deep learning approaches: A case study on the Niagara tunnel project. Undergr. Space.

